# Cluster-randomised trial evaluating a complex intervention to improve mental health and well-being of employees working in hospital – a protocol for the SEEGEN trial

**DOI:** 10.1186/s12889-019-7909-4

**Published:** 2019-12-17

**Authors:** Nadine Mulfinger, Anja Sander, Felicitas Stuber, Regina Brinster, Florian Junne, Ronald Limprecht, Marc N. Jarczok, Tanja Seifried-Dübon, Monika A. Rieger, Stephan Zipfel, Martin Peters, Maja Stiawa, Imad Maatouk, Madeleine Helaß, Christoph Nikendei, Eva Rothermund, Nicole Hander, Ute Ziegenhain, Manuela Gulde, Melanie Genrich, Britta Worringer, Janna Küllenberg, Karl Blum, Stefan Süß, Elena Gesang, Sascha Ruhle, Contributors of the SEEGEN consortium, Anja Greinacher, Kirsten Bikowski, Marieke Born, Antonia Drews, Lina Hermeling, Bernd Puschner, Elena Tsarouha, Lucia Jerg-Bretzke, Meinhard Kieser, Andreas Müller, Jochen Schweitzer-Rothers, Peter Angerer, Harald Gündel

**Affiliations:** 1 Clinic of Psychosomatic Medicine and Psychotherapy, University Hospital Ulm, Albert-Einstein-Allee 23, 89081 Ulm, Germany; 2 Institute of Medical Biometry and Informatics, University of Heidelberg, Im Neuenheimer Feld 130.3, 69120 Heidelberg, Germany; 3 Department of Psychosomatic Medicine and Psychotherapy, University Hospital Tübingen Osianderstraße 5, 72076 Tübingen, Germany; 4 Institute for Occupational and Social Medicine and Health Services Research, University Hospital Tübingen, Wilhelmstraße 27, 72074 Tübingen, Germany; 5 Department of Psychiatry II, Ulm University and Bezirkskrankenhaus Günzburg, Ludwig-Heilmeyer-Str. 2, 89312 Günzburg, Germany; 6 Department for General Internal Medicine and Psychosomatics, University Hospital Heidelberg, Im Neuenheimer Feld 410, 69120 Heidelberg, Germany; 7 Clinic of Child- and Adolescents Psychiatry / Psychotherapy, University Hospital Ulm, Steinhövelstraße 3, 89075 Ulm, Germany; 8 Institute of Psychology, Work and Organisational Psychology, University of Duisburg-Essen, Universitätsstr. 2, 45141 Essen, Germany; 9 Institute of Occupational and Social Medicine, Heinrich-Heine-University Düsseldorf, Universitätsstraße 1, 40225 Düsseldorf, Germany; 10 Institute for Medical Psychology, University Hospital Heidelberg, Bergheimer Straße 20, 69115 Heidelberg, Germany; 11 German Hospital Institute (DKI) e.V, Hansaallee 201, Haus 1, 40549 Düsseldorf, Germany; 12 Chair of Business Administration, in particular Work, Human Resource Management and Organisation Studies, Heinrich-Heine-University Düsseldorf, Universitätsstraße 1, 40225 Düsseldorf, Germany

**Keywords:** Mental health, Hospital, Work-related psychological stress and strain, Complex intervention, Cluster-randomised controlled trial; waitlist control, Health care workers, Well-being

## Abstract

**Background:**

Health care employees in Germany and worldwide are exposed to a variety of stressors. However, most of the hospitals in Germany lack a systematic workplace health management. Thus, this study aims at the evaluation of the effects of a behavioural as well as organisational (´complex´) intervention on the mental health and well-being of hospital staff.

**Methods:**

Mental health in the hospital workplace (SEElische GEsundheit am Arbeitsplatz KrankeNhaus – SEEGEN) is an unblinded, multi-centred cluster-randomised open trial with two groups (intervention group (IG) and waitlist control group (CG)). Study participants in the intervention clusters will receive the complex intervention; study participants in the waitlist control clusters will receive the complex intervention after the last follow-up measurement. The intervention consists of five behavioural and organisational intervention modules that are specifically tailored to hospital employees at different hierarchical and functional levels. Hospital staff may select one specific module according to their position and specific needs or interests. Towards the end of the intervention roundtable discussions with representatives from all professional groups will be held to facilitate organisational change. Primary outcome is the change in emotional and cognitive strain in the working environment, from baseline (T0) to 6 month-follow up (T1), between IG and CG. In addition, employees who do not participate in the modules are included in the trial by answering shorter questionnaires (cluster participants). Furthermore, using mixed methods, a process evaluation will identify uptake of the intervention, and mediators and moderators of the effect.

**Discussion:**

There seems to be growing psychological strain on people working in the health care sector worldwide. This study will examine whether investing directly in the hospital staff and their interpersonal relationship may lead to measurable benefits in subjective well-being at the workplace and improved economic performance indicators of the hospital. In case of a positive outcome, health promotion strategies looking at behavioural as well as organisational components within the hospital may gain additional importance, especially in regard of the growing financial pressure within the health sector.

**Trial registration DRKS:**

The SEEGEN study is registered at the German Clinical Trial Register (DRKS) under the DRKS-ID DRKS00017249. Registered 08 October 2019, URL.

https://www.drks.de/drks_web/navigate.do?navigationId=trial.HTML&TRIAL_ID=DRKS00017249.

## Background

Mental health as well as the development and progress of mental diseases are influenced by biological (e.g., genetic predispositions), psychological (e.g., early childhood trauma), and social factors [1]. Many of the social factors can be localised in the working environment. On the one hand, in a positive sense, for example, the experience of recognition, the opportunity for being creative and productive, or the establishment of social contacts, all can help to cope with stressful situations. On the other hand, in a negative sense, for example when the modern, complex world of work is very demanding and, thus, might create feelings of excess strain or isolation. Work stress can therefore have a negative impact both on the individual (e.g., mental disease like depression, [2]; somatic disease like cardiovascular diseases, [3] and its associated risk markers [4]) and on the organisation (e.g. absenteeism, job dissatisfaction, [5]; for an overview on work stress models see for example [6]).

Hospital employees are a particularly vulnerable group in terms of endangered health because of their high occupational psychological stress levels [7]. Demographic change, a growing and often painful lack of adequately trained staff and reduced subsidies for hospitals add to this problem [8]. As one German occupational health physician puts it: “Far beyond the scope of what other organisations demand from their members, hospitals expect their personnel to be altruistic, devoted, and orientated towards ethical and moral principles. In this respect, the organisation is reflecting the values that have already been incorporated by its members, strengthens them and in turn expects them from every single person” [9]. The demanding working environment in a hospital is one factor which explains the high levels of perceived psychological stress of hospital employees [7]. In a systematic review [7], the individual and organisational impact of work-related stress in the Australian and international health and social services (HCS) sectors were examined. The results demonstrated high levels of occupational stress in the HCS sector. Apart from work-related stress, employees working in hospitals even showed an increased number of suicides [10].

The above-mentioned ethical and moral principles the hospitals expect from their employees should, from a humanitarian perspective, also apply to the hospital as the employer. In addition, from a legal perspective, since 2013 the German law also obliges employers to the assessment, maintenance, and fostering of mental health at the workplace through an amendment to the German occupational health and safety act [11].

Behavioural and organisational measures in order to maintain and foster mental health for health care workers have already been implemented and evaluated [12]. However, to date, preventive actions within the hospital only targeted either single professional groups (e.g., nurses in Müller et al., 2018 [13]) or subareas/organisational subunits within a hospital [14] and not the whole hospital, or, they utilized either behavioural or organisational preventive measures, but not a *complex intervention* comprising both prevention types. A review by Ruotsalainen et al., 2015 [12] emphasizes the need for methodologically high-quality studies and the evaluation of complex interventions which combine behavioural and organisational interventions in the health care system. This gap is presumably due to the specific multidimensional complexity of hospital structure.

In this vein, most of the hospitals, at least in Germany, lack a systematic workplace health management which combines behavioural as well as organisational prevention, and they also lack the inclusion of these variables in their overall economic evaluation [8]. Although, in other sectors, some studies indicate an increased effectiveness of the combination of behavioural and organisational intervention types [15].

Against this background, the purpose of this study is to examine whether a multi-centred complex intervention consisting of combined behavioural and organisational interventions for different target groups in hospitals (e.g. leaders, staff members of differed professional groups) called *Mental health in the hospital workplace* (SEElische GEsundheit am Arbeitsplatz KrankeNhaus, SEEGEN) can improve organisational policies, practices, and procedures to protect workers mental health and safety.

In general, mental health can be defined “as a state of well-being in which every individual realizes his or her own potential, can cope with the normal stresses of life, can work productively and fruitfully, and is able to make a contribution to her or his community.” [16]. Beyond this definition mental health can be seen as a continuum ranging from a negative states of mental health (e.g., the presence of depression or burnout) to positive mental health states (e.g., the presence of well-being [17];).

To do justice to the complex construct of mental health and the multi-level structure of hospitals, we decided to operationalize mental health in this study through different constructs on individual/participant level as well as on hospital/clinic level. In order to depict this approach, the three utilized constructs of mental health will be briefly introduced. First, we consider the concept of *irritation* [18]. This individual-based construct comprises the two sub dimensions of cognitive strain (e.g., distance oneself from work) and emotional strain (e.g., increased negative social interaction) at the workplace in the so-called Irritation Scale (IRR). Irritation can be seen as a sensitive measure, which captures individual mental well-being in the work context. Second, at a more general individual level, we consider *subjective well-being* [19], which is not as closely connected to the workplace as irritation, and therefore a broader concept. Finally, at the hospital level, we consider the *psychosocial safety climate* (PSC [20]) which refers to organisational policies, practices, and procedures to protect workers mental health and safety [21]. A high level of PSC can be argued as a fundament of mental health preventive working conditions [22].

**Fig. 1:**
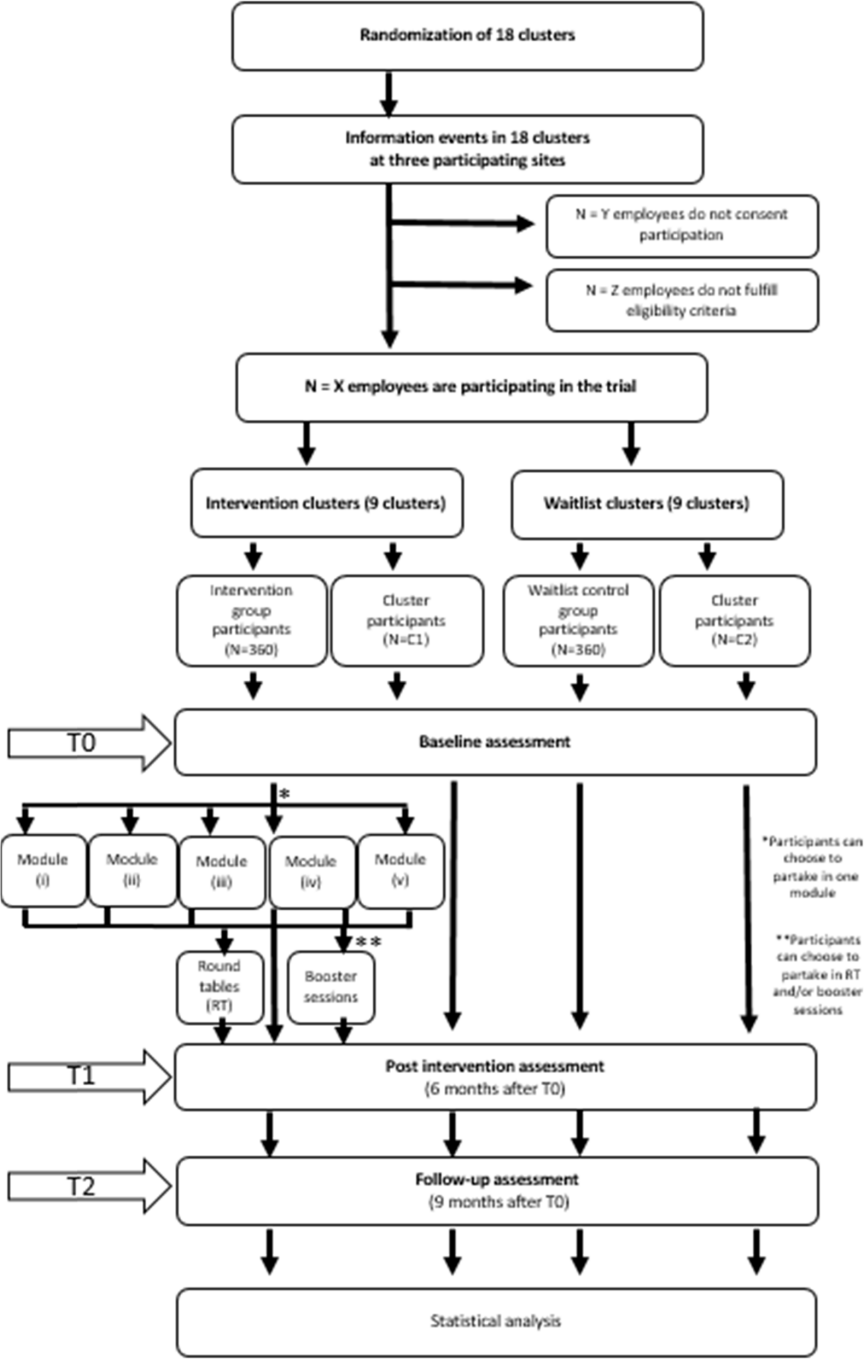
Flow diagram

To sum up, the SEEGEN trial will evaluate the effect of a complex intervention on mental health and well-being of hospital employees at different levels of hierarchy and functional areas. It is designed as a multi-centred cluster-randomised open trial with a waitlist control group. For a flow diagram of the trial design, see Fig. 1. This protocol has been drafted in accordance with the SPIRITguidelines (see Table 1; [23]).

**Table 1:** SPIRIT 2013 Checklist: Recommended items to address in a clinical trial protocol and related documents^a^

Section/item	Item No	Description	Addressed on page number
Administrative information			
Title	1	Descriptive title identifying the study design, population, interventions, and, if applicable, trial acronym	1
Trial registration	2a	Trial identifier and registry name. If not yet registered, name of intended registry	2
	2b	All items from the World Health Organization Trial Registration Data Set	not yet available
Protocol version	3	Date and version identifier	3; 2
Funding	4	Sources and types of financial, material, and other support	15
Roles and responsibilities	5a	Names, affiliations, and roles of protocol contributors	1; 15
	5b	Name and contact information for the trial sponsor	15
	5c	Role of study sponsor and funders, if any, in study design; collection, management, analysis, and interpretation of data; writing of the report; and the decision to submit the report for publication, including whether they will have ultimate authority over any of these activities	15
	5d	Composition, roles, and responsibilities of the coordinating centre, steering committee, endpoint adjudication committee, data management team, and other individuals or groups overseeing the trial, if applicable (see Item 21a for data monitoring committee)	13
Introduction			
Background and rationale	6a	Description of research question and justification for undertaking the trial, including summary of relevant studies (published and unpublished) examining benefits and harms for each intervention	2-3
	6b	Explanation for choice of comparators	-
Objectives	7	Specific objectives or hypotheses	3
Trial design	8	Description of trial design including type of trial (eg, parallel group, crossover, factorial, single group), allocation ratio, and framework (eg, superiority, equivalence, noninferiority, exploratory)	3; 11
Methods: Participants, interventions, and outcomes			
Study setting	9	Description of study settings (eg, community clinic, academic hospital) and list of countries where data will be collected. Reference to where list of study sites can be obtained	3
Eligibility criteria	10	Inclusion and exclusion criteria for participants. If applicable, eligibility criteria for study centres and individuals who will perform the interventions (eg, surgeons, psychotherapists)	6
Interventions	11a	Interventions for each group with sufficient detail to allow replication, including how and when they will be administered	6-8
	11b	Criteria for discontinuing or modifying allocated interventions for a given trial participant (eg, drug dose change in response to harms, participant request, or improving/worsening disease)	13
	11c	Strategies to improve adherence to intervention protocols, and any procedures for monitoring adherence (eg, drug tablet return, laboratory tests)	13
	11d	Relevant concomitant care and interventions that are permitted or prohibited during the trial	-
Outcomes	12	Primary, secondary, and other outcomes, including the specific measurement variable (eg, systolic blood pressure), analysis metric (eg, change from baseline, final value, time to event), method of aggregation (eg, median, proportion), and time point for each outcome. Explanation of the clinical relevance of chosen efficacy and harm outcomes is strongly recommended	8-11
Participant timeline	13	Time schedule of enrolment, interventions (including any run-ins and washouts), assessments, and visits for participants. A schematic diagram is highly recommended (see Figure)	11
Sample size	14	Estimated number of participants needed to achieve study objectives and how it was determined, including clinical and statistical assumptions supporting any sample size calculations	3
Recruitment	15	Strategies for achieving adequate participant enrolment to reach target sample size	11
Methods: Assignment of interventions (for controlled trials)			
Allocation			
Sequence generation	16a	Method of generating the allocation sequence (eg, computer-generated random numbers), and list of any factors for stratification. To reduce predictability of a random sequence, details of any planned restriction (eg, blocking) should be provided in a separate document that is unavailable to those who enrol participants or assign interventions	11
Allocation concealment mechanism	16b	Mechanism of implementing the allocation sequence (eg, central telephone; sequentially numbered, opaque, sealed envelopes), describing any steps to conceal the sequence until interventions are assigned	11
Implementation	16c	Who will generate the allocation sequence, who will enrol participants, and who will assign participants to interventions	11
Blinding (masking)	17a	Who will be blinded after assignment to interventions (eg, trial participants, care providers, outcome assessors, data analysts), and how	11
	17b	If blinded, circumstances under which unblinding is permissible, and procedure for revealing a participant’s allocated intervention during the trial	-
Methods: Data collection, management, and analysis			
Data collection methods	18a	Plans for assessment and collection of outcome, baseline, and other trial data, including any related processes to promote data quality (eg, duplicate measurements, training of assessors) and a description of study instruments (eg, questionnaires, laboratory tests) along with their reliability and validity, if known. Reference to where data collection forms can be found, if not in the protocol	8-11
	18b	Plans to promote participant retention and complete follow-up, including list of any outcome data to be collected for participants who discontinue or deviate from intervention protocols	11
Data management	19	Plans for data entry, coding, security, and storage, including any related processes to promote data quality (eg, double data entry; range checks for data values). Reference to where details of data management procedures can be found, if not in the protocol	11-13
Statistical methods	20a	Statistical methods for analysing primary and secondary outcomes. Reference to where other details of the statistical analysis plan can be found, if not in the protocol	12-13
	20b	Methods for any additional analyses (eg, subgroup and adjusted analyses)	12-13
	20c	Definition of analysis population relating to protocol non-adherence (eg, as randomised analysis), and any statistical methods to handle missing data (eg, multiple imputation)	13
Methods: Monitoring			
Data monitoring	21a	Composition of data monitoring committee (DMC); summary of its role and reporting structure; statement of whether it is independent from the sponsor and competing interests; and reference to where further details about its charter can be found, if not in the protocol. Alternatively, an explanation of why a DMC is not needed	13
	21b	Description of any interim analyses and stopping guidelines, including who will have access to these interim results and make the final decision to terminate the trial	13
Harms	22	Plans for collecting, assessing, reporting, and managing solicited and spontaneously reported adverse events and other unintended effects of trial interventions or trial conduct	13
Auditing	23	Frequency and procedures for auditing trial conduct, if any, and whether the process will be independent from investigators and the sponsor	13
Ethics and dissemination			
Research ethics approval	24	Plans for seeking research ethics committee/institutional review board (REC/IRB) approval	15
Protocol amendments	25	Plans for communicating important protocol modifications (eg, changes to eligibility criteria, outcomes, analyses) to relevant parties (eg, investigators, REC/IRBs, trial participants, trial registries, journals, regulators)	15
Consent or assent	26a	Who will obtain informed consent or assent from potential trial participants or authorised surrogates, and how (see Item 32)	13
	26b	Additional consent provisions for collection and use of participant data and biological specimens in ancillary studies, if applicable	-
Confidentiality	27	How personal information about potential and enrolled participants will be collected, shared, and maintained in order to protect confidentiality before, during, and after the trial	13
Declaration of interests	28	Financial and other competing interests for principal investigators for the overall trial and each study site	15
Access to data	29	Statement of who will have access to the final trial dataset, and disclosure of contractual agreements that limit such access for investigators	13
Ancillary and post-trial care	30	Provisions, if any, for ancillary and post-trial care, and for compensation to those who suffer harm from trial participation	13
Dissemination policy	31a	Plans for investigators and sponsor to communicate trial results to participants, healthcare professionals, the public, and other relevant groups (eg, via publication, reporting in results databases, or other data sharing arrangements), including any publication restrictions	13
	31b	Authorship eligibility guidelines and any intended use of professional writers	15
	31c	Plans, if any, for granting public access to the full protocol, participant-level dataset, and statistical code	-
Appendices			
Informed consent materials	32	Model consent form and other related documentation given to participants and authorised surrogates	Supplementary material
Biological specimens	33	Plans for collection, laboratory evaluation, and storage of biological specimens for genetic or molecular analysis in the current trial and for future use in ancillary studies, if applicable	-

^a^It is strongly recommended that this checklist be read in conjunction with the SPIRIT 2013 Explanation & Elaboration for important clarification on the items. Amendments to the protocol should be tracked and dated. The SPIRIT checklist is copyrighted by the SPIRIT Group under the Creative Commons “Attribution-NonCommercial-NoDerivs 3.0 Unported” license.

### Aims and objectives

Specific objectives are to

1. evaluate whether a complex intervention consisting of behavioural and organisational preventive elements leads to an improvement in the subjectively perceived mental health of hospital employees at an individual as well as at hospital level.
2. evaluate whether the intervention also has an effect on other, e.g., economic variables.
3. identify uptake of the intervention, and mediators and moderators of the effect by means of process analysis and mixed-methods approach.

## Methods

### Participants, interventions and outcomes

#### Study setting

The study takes place in three hospitals each representing one specific type of hospital within the German hospital setting: a hospital, which is owned by a private health company, a community hospital and a university hospital.

#### Study population

All employees in the participating units of the three study locations will be potential participants. At least six clusters are planned per location, with altogether about 720 potential participants (360 participants in intervention- and 360 participants in waitlist control group). Figure 1 shows the flow chart of enrolment, baseline and post intervention measurements and follow-up in the SEEGEN trial.

#### Sample size

The sample size calculation is based on the primary endpoint, i.e. the absolute change in the total score of the Irritation Scale [18] from T0 to T1, which will be evaluated by an ANCOVA approach. The sample size is calculated based on a two-sided two sample t-test with two-sided significance level α = 0.05 and power 1-β = 0.8, which provides a conservative estimate for the necessary sample size for the ANCOVA evaluation. The intervention is assumed to yield a medium effect, hence an effect size of d = 0.4 is used for sample size calculation. These assumptions yield a sample size of 100 participants per group (200 for both intervention and control). Taking into account the dependencies within clusters and simultaneously being conservative via assuming a high intraclass correlation coefficient of ICC = 0.05 and a mean cluster size of about 40 participants yields a design effect of 1 + 0.05*(40–1) = 2.95. Therefore, the necessary sample size is 2.95*200 ≈ 590 participants. Furthermore, a drop-out rate of 18% will be considered. Taken together, this amounts to a total sample size of 720 participants in 18 clusters. Under consideration of possible cluster drop-outs, more than 18 clusters might be recruited which will lead to an increased power. The sample size was calculated using the software SAS 9.4 (SAS Institute, Heidelberg, Germany).

#### Eligibility criteria

Inclusion criteria for study locations are:

- Willingness to participate in the study regardless of randomisation to the intervention or waitlist control arm
- Willingness to complete three questionnaires (applicable to intervention group, waitlist control group and cluster participants)

Eligible criteria for employees who want to take part in the trial are:

1. Age: 18–70 years old,
2. Written informed consent,
3. Sufficient German language skills to fill out the questionnaires

### Intervention

#### Intervention overview

The complex intervention is built on experiences from five previous pilot projects carried out between 2017 and 2019. These prior pilots operate on both, the behavioural and the organisational levels, and will be used as a combined stress-mitigating additive during the complex 9-month intervention period in three locations including clusters from small regional hospitals to university hospitals with maximum clinical care. Five individual modules with different thematic priorities will be offered to hospital employees: (i) Top Management Training, (ii) Promoting Stress Preventive Relational Leadership Competence, (iii) Dilemma Competency – Coping by Taking Responsibility, (iv) Reconciling Work and Family Life and (v) Stay Healthy at Work. Importantly and in contrast to other studies, employees may choose one module according to their specific needs and interests depending on their position in the hospital. The maximum duration of one module will be 12 h, the minimum duration 6 h.

#### Intervention modules

(i) Top Management Training

In the overall concept, this sub-project represents a kick-off workshop addressed to clinic managers of medicine and nursing. This workshop focusses on sensitizing senior nurses and senior physicians for the topic of healthy work design, and prepares the ground for the other approaches of the complex intervention.

At the beginning of the workshop, the overall concept of the SEEGEN study is presented and the most urgent stressors and resources in the respective departments of the participants are queried. This is followed by a presentation on the current scientific status regarding the relationships between working conditions and employee health, based on the four most prominent models (job-demand control model, effort-reward imbalance, organisational justice, and social support [2, 24–26]) as well as the relationships between working conditions and quality of treatment, cost-effectiveness, and employer attractiveness. In addition, the difference between behavioural and organisational-preventive measures is explained and elaborated based on the presentation of the other interventions of the SEEGEN study. The participants are introduced to a 4-step plan for the implementation of preventive measures. The first step is to discuss options for carrying out a psychosocial risk assessment (which is mandatory according to the German occupational health and safety act), the second step to discuss the verbalisation and prioritisation of change goals, the third step to draw up a plan of action, and the last step to develop options for evaluation and performance review. This draws attention to the round tables following each of the other intervention modules. The participants should be motivated to participate in the round tables as well as to motivate their employees to participate in the further interventions of the SEEGEN study.

(ii) Promoting Stress Preventive Relational Leadership Competence

Leadership behaviour has been described as an important variable for employees’ psychological well-being (e.g. [17]). Since a hospital can be perceived as a psychologically demanding workplace for employees (consider for example daily interaction with seriously ill as well as dying patients and bereaved family members, staff shortage, and economical pressure) it is not only staff members’ own responsibility but also an important leaders’ task to preserve and foster staff members’ mental health. Therefore, we provide a leadership group module, which concentrates on relational and stress preventive aspects of leadership. It is open for leaders of middle management level in all occupational groups. The leadership module with a total duration of 12 hours consists of four parts each lasting 3 hours. Part 1 and 2 of the module will be implemented on 1 day, part 3 and 4 on another one. The interval between Day 1 and Day 2 (approximately 3 weeks) shall be used by participants to implement their new knowledge and techniques into their daily work situation (Practical Phase). Every part comprises theoretical parts (e.g., theoretical input through frontal presentations, instructional videos) to generate new knowledge as well as practical and interactive parts (e.g., group discussions, role-plays, single work or partner work) to discuss and transfer this knowledge into the everyday work. Contentwise the focus lies on: Part1. Competent handling of the leaders’ own stress as a prerequisite for stress preventive leadership, Part 2. Health oriented and stress-preventive leadership models including Transformational Leadership (e.g. 27, 28) and the Leader – Member-Exchange Model of Leadership (LMX, for an overview see [29]) to establish successful working relations [30], Part 3. Motives, needs and specific stressors of staff members and the requirements to fostering a mindful communication and Part 4. Understanding social dynamics in teams at the complex workplace hospital and enabling a constructive team culture as a leader.

(iii) Dilemma Competency – Coping by Taking Responsibility

Employees are confronted with and are expected to handle contradictions and ambivalent decision making processes in organisations [31]. If they fail to face these in a constructive way, they are at heightened risk to experience mental strains (e.g. [32]). To support employees working in the public health sector to meet the working conditions, an already successfully implemented training [33] on dilemma competency has been adapted to the workspace hospital.

The aim of the module is to provide the participants with mental and action-related abilities to understand contradictory demands in their organisation and to meet associated demands constructively through negotiation and decision-making processes. Each group intervention consists of 2 workshop days (à 6 h) and is administered over a period of maximum 8 weeks.

Overall, this element of the complex intervention strives for prevention of stress-related diseases and the promotion of an enhanced sense of meaningfulness experienced by the participants. In times of an increasing aggravation of working conditions due to economic strain, competitive constraints and shortage of skilled employees in the health care system, dilemma management supports employees in dealing with daily work strains.

(iv) Reconciling Work and Family Life

This intervention module aims to improve the currently challenging situation of hospital employees within the family phase, i.e. raising children. The one-day workshop consists of four sessions of 90 min each. Aim of the module is to improve one’s ability to cope with reconcilability stress by enabling a personal analysis and reflection of each participant’s situation. This analysis of the professional and family environment is guided by worksheets developed for the module that are worked on individually or in groups. Further elements are the subject-related impulse lectures regarding the connection between stress experience, stress response and the influence of personal stress on the relationship to the child as well as developmental psychological findings for practical everyday life as a parent. The participants are instructed on practical stress management by yoga. In contrast to a pure coping workshop, this module is also highlighting the constellation “dilemma” in the context of a role-play, in order to work out how to deal with an unsatisfactorily solvable compatibility situation in a further step.

(v) Stay Healthy at Work

This module deals with healthy ageing in the professional health care context. It was developed from a pilot project at the Medical Hospital of the University of Heidelberg, which promotes mental health and the associated work ability of caregivers of advanced working age [34]. The aim is to develop a behavioural preventive measure to promote healthy ageing in 6 sessions spread over 1.5 days. The trainer especially promotes interaction between the participants and integrates resources.

On the first day, 4 sessions are worked on: After getting to know each other in session one, the participants reflect on individual stressors as well as cognitive and palliative coping strategies in the second session. In the third session, aging is not only considered as a stressor but also established as a resource. The central topic of the first day is the ‘Theory of Selection, Optimization and Compensation (SOC)’, which is presented to the participants in the fourth session. This approach includes a resource-oriented focus and optimal use of resources despite increasing restrictions due to ageing. After theoretical input, the participants are guided to develop personal projects in their working environment. They begin with the concrete planning and implementation of the SOC approach in their individual projects. Between the first and the second workshop day there is a time period of several weeks for participants to test their project related aims and strategies.

The second workshop day focuses on the reflection of SOC projects of the participants. In the fifth session, the participants present the status of their project. In the last session a summary, take-home message, and conclusion of the training takes place.

#### Roundtable discussions

In addition to the modules, roundtable discussions will be offered to facilitate organisational change and develop concrete proposals for action. There will be one round table per module type. Five round tables will be offered at each study location. The roundtables are a participation instrument in which representatives of different positions and interests come together under a neutral moderation in order to process various suggestions for improvement made by employees during the modules. As a first step, concrete measures operating on the organisational level will be developed (so-called 1st-order round tables), which will be presented in a second step to the decision-makers at each study location (including the Executive Board; so-called 2nd-order round tables). The duration per round table is 2–4 h.

#### Booster sessions

All of the modules (except for the Top Management Training) will be followed by one 2 h booster session to enhance training effects. In these booster sessions, the exchange of transfer experiences from training into daily hospital life is enabled. It is hoped that some participants will continue to exchange experiences in a self-organising fashion, even after project termination. Booster sessions will be offered after all modules have finished, before data monitoring at T1, during the same month when 1st order round tables take place. For all intervention clusters, this should be May 2020. Participants are invited to join the booster sessions specific for their chosen module.

At the end of the SEEGEN trial, a discussion with key players of the German health system will take place. The discussion aims at the implementation of the project results into the health system and health organisations like hospitals. For this purpose, focus groups and interviews with representatives of the political system, health insurances, hospital associations and staff associations are planned. Main topics are the following:

- Appraisal of the developed programs for work place health management
- Critical factors for implementation
- Contribution by management and leadership to work place health promotion
- Need of law revisions
- Need of further development of standards for working conditions

### Outcomes

In the following section, details of all assessments used in the SEEGEN trial are described.

#### Primary outcome

The outcome will be the change in the total score of the IRR [18] from baseline (T0) to 6 months after baseline (T1) compared between IG and CG. The eight-item IRR assesses the emotional and cognitive strain in the working environment. Items are measured on a 7-point Likert scale, ranging from 1 “not at all” to 7 “almost completely correct”.

#### Secondary outcomes

##### Main secondary outcomes

- Change in the World-Health Organization Well-Being Index (WHO-5 [19]) from T0 to T1 between the IG and CG.
  Subjective psychological well-being will be measured using the five-item World Health Organization Well-Being Index (WHO-5). Items are measured on a 6-point Likert scale, ranging from 0 “at no time” to 5 “all of the time”. The raw score ranging from 0 to 25 is multiplied by 4 to give the final score ranging from 0 representing the worst well-being to 100 representing the best well-being.
- Change in Psychosocial Safety Climate (PSC-12 [20]) from T0 to T1 between the IG and CG.
  Psychosocial safety climate will be measured using the 12-item Psychosocial Safety Climate Scale [20]. The PSC-12 consists of four dimensions: (1) organisation participation (3 items), (2) organisation communication (3 items), (3) management priority (3 items) and (4) management commitment (3 items). Items are measured on a 5-point Likert scale, ranging from 1 “strongly disagree” to 5 “strongly agree”.

### Further secondary outcomes

Change in the total score of the IRR from T0 to 9 months after baseline (T2) as well as the changes from T0 to T1 and T0 to T2 for both subscales between the IG and CG.

The change in scores from T0 to T1, as well as from T0 to T2, between the IG and CG of the following:

- World Health Organization Well-Being Index (WHO-5 Well-Being Index)
- Psychosocial Safety Climate (PSC-12)
- Effort-Reward Imbalance Scale – Short version (ERI [35];).
  Effort will be measured by three items (ERI1-ERI3). Items are measured on a 4-point Likert scale, ranging from 1 “strongly disagree” to 4 “strongly agree” with higher sum score between 3 and 12 indicating more perceived efforts. Reward is measured by seven items (ERI4-ERI10). A low sum score of these items between 7 and 28 represents fewer perceived occupational rewards.
- Work Analysis Instrument for Hospitals – Self-Report Version (Tätigkeits- und Arbeitsanalyseverfahren für das Krankenhaus; TAA-KH-S [36];)
  Two subscales of a German questionnaire for hospital employees will be used to measure their working conditions: (1) job decision authority (shortened from 9 to 3 items); (2) quantitative job demands (3 items). Items are measured on a 5-point Likert scale, ranging from 1 “no, not at all” to 5 “yes, absolutely”.
- Questionnaire on Integrative Leadership (Fragebogen zur Integrativen Führung, FIF [28])
  The questionnaire on integrative leadership is a standardized instrument, which records leadership and communication style in four modules (module A: transformational and transactional leadership, module B: instrumental leadership, module C: communication and module D: negative leadership). In this study, module A in its self-assessment version for leaders and its external assessment version for employees will be used. The multidimensional construct of transformational leadership consists of six core behaviours (“fostering innovation”, “team spirit development”, “performance development”, “individuality focus”, “providing a vision” and “being a role model”). Participants have to rate 24 statements at a five point Likert-scale from 1 “agree not at all” to 5 “totally agree”. The items’ ratings can be summarized to 10 different scale scores or to one overall score each (transformational leadership, transactional leadership and negative leadership). The scales of transformational leadership show a sufficient intern consistency with Cronbach’s α = .79–.92 for the external assessment and Cronbach’s α = .71–.83 for the self-assessment [28].
- Short version of the Occupational Self-Efficacy Scale (SOSES [37];)
  Occupational self-efficacy will be measured using the Short version of the Occupational Self-Efficacy Scale. The instrument consists of 6 items rated on a six-point Likert scale ranging from 1 (not at all true) to 6 (completely true) with higher values reflecting higher occupational self-efficacy.
- Top Management Evaluation Sheet
  At the beginning and at the end of the module *Top Management Training*, participants will be asked to complete an evaluation form consisting of 28 items. The questions relate to measures to promote the mental health of employees at the hospital (e.g. offers for stress prevention, activities to improve working conditions) and the role of the management in this issue.
- Organisational indicators on individual/participant level:
  - Job satisfaction ([38]; 8 items, measured on different 5-point Likert scales, ranging from 1 “wrong” to 5 “right”, 1 “not at all interesting” to 5 “yes, very interesting”, 1 “very few opportunities” to 5 “yes, a lot of opportunities”, 1 “very discontent” to 5 “yes, very content”, 1 “definitely not” to 5 “yes, for sure”)
  - Employer attractiveness ([39]; 5 items, measured on a 7-point Likert scale, ranging from 1 “strongly disagree” to 7 “strongly agree”)
  - Presenteeism ([40]; 1 item, measured on a 5-point scale; “never”, “seldom”, “occasionally”, “frequently”, “very often”)
  - Intention to leave ([41]; 1 item, measured on a 5-point scale; “never”, “a few times a year”, “a few times a month”, “a few times a week”, “everyday”)
  - Recommendation (modified version of Reichheld, 2003 [42]; “How likely is it that you would recommend this company to a friend or colleague?“, measured on an 11-point Likert scale, ranging from 1 “highly unlikely” to 11 “highly likely”)
  - Absenteeism (modified version of Caverley et al., 2007 [43]; “Please estimate, how many days on average per month you are being absent due to an illness.”)
  - Work overtime (“How many hours have you worked overtime in the past month?”)
  - Working hours (“What is your contractually agreed working time per week?”, “What is your average working time per week?”)
  - Economic situation of clinic/unit (adapted version of Hall & Rohrbach-Schmidt, 2013 [44], “How do you assess the economic situation of the clinic/unit you work at?”, measured on a 4-point Likert scale, ranging from 1 “very good” to 4 “bad”, with 5 “don’t know”).
  - Job security ([45], “My job security is good”, measured on a 4-point Likert scale, ranging from 1 “strongly disagree” to 4 “strongly agree”).
  - Cooperation between occupational groups ([36], shortened from 5 to 2 items, TAA-KH-S subscale social climate, measured on a 5-point Likert scale, ranging from 1 “no, not at all” to 5 “yes, absolutely”).
  Moreover, some organisational indicators on hospital/clinic level will be collected on a regular basis via request from the hospitals accounting division, depending on the availability. These organisational indicators will be assigned to the clusters, if possible, depending on the level of detail of the data and the homogeneity/heterogeneity of the clusters.
  l. Turnover ($\mathrm{turnover}\ \mathrm{rate}=\frac{\mathrm{number}\ \mathrm{of}\ \mathrm{employees}\ \mathrm{who}\ \mathrm{left}\ \mathrm{in}\ \mathrm{year}\ \mathrm{x}}{\mathrm{total}\ \mathrm{number}\ \mathrm{of}\ \mathrm{employees}\ \mathrm{at}\ \mathrm{the}\ \mathrm{beginning}\ \mathrm{of}\ \mathrm{year}\ \mathrm{x}}\ \mathrm{x}\ 100$,number of applicants, number of persons hired, number of persons retired, number of dismissals, number of expiring fixed-term contracts)
  m. $$ \mathrm{Absence}\ \mathrm{rate}=\frac{\mathrm{absence}\ \left(\mathrm{in}\ \mathrm{hours}\right)\ \mathrm{in}\ \mathrm{year}\ \mathrm{x}}{\mathrm{gross}\ \mathrm{working}\ \mathrm{time}\ \left(\mathrm{number}\ \mathrm{of}\ \mathrm{employees}\ast \mathrm{working}\ \mathrm{hours}\ \mathrm{per}\ \mathrm{week}\right)\ \mathrm{in}\ \mathrm{year}\ \mathrm{x}}\ \mathrm{x}\ 100 $$
  n. Work overtime rate = $\frac{\mathrm{work}\ \mathrm{overtime}\ \left(\mathrm{in}\ \mathrm{hours}\right)\ \mathrm{in}\ \mathrm{year}\ \mathrm{x}}{\mathrm{contracted}\ \mathrm{working}\ \mathrm{time}\ \left(\mathrm{in}\ \mathrm{hours}\right)\ \mathrm{in}\ \mathrm{year}\ \mathrm{x}}\ \mathrm{x}\ 100$
  o. $$ \mathrm{Sickness}\ \mathrm{absence}\ \mathrm{rate}=\frac{\mathrm{sickness}\ \mathrm{absence}\ \left(\mathrm{in}\ \mathrm{hours}\right)\ \mathrm{in}\ \mathrm{year}\ \mathrm{x}}{\mathrm{planned}\ \mathrm{working}\ \mathrm{time}\ \left(\mathrm{in}\ \mathrm{hours}\right)\ \mathrm{in}\ \mathrm{year}\ \mathrm{x}}\ \mathrm{x}\ 100 $$
  p. Age distribution = number of employees sorted by age classes

In addition, cluster participants (no module participation) will be asked to complete a shorter questionnaire at T0, T1 and T2 consisting of the following measurements:

- IRR [18], as described above,
- WHO-5 [19], as described above and
- PSC-12 [20], as described above,
- Global Transformational Leadership Scale (GTL [46];)
  To keep the questionnaire for cluster participants short, the Questionnaire on Integrative Leadership (FIF [28]) was replaced by the GTL, which is a 7-item questionnaire to measure transformational leadership [27] from 1 “to a very small extent” to 5 “to a very large extent”. Higher scores indicate higher transformational leadership behaviour. The German version of the questionnaire was already used in Rigotti et al., 2014 ([47] p.67) the same translation will be used here.

Based on single items, cluster participants will be asked about their perception of the needs and benefits (personal and for the units they work at) of the module-offer, too. The outcome will be the change in the scores of IRR, WHO-5, PSC-12, GTL and single items from T0 to T1, as well as from T0 to T2.

### Process evaluation

A thorough process evaluation using mixed-methods will be an integral part of this trial. First, focus groups [48] (about *N* = 8 per group) and individual interviews (*n* = 12) will be conducted with a subsample of participants from all study sites before and after the implementation of the intervention to identify participants’ attitudes, perceptions, and experiences regarding the intervention. Focus groups will be conducted with clinic staff without management responsibilities and those in middle management positions. Individual interviews will be conducted with participants in higher management positions (for example heads of department). While focus groups will take place at participants’ workplace, individual interviews will be conducted on-site or via phone or video call. Group discussions and interviews will be recorded and transcribed. All transcripts will be analysed using qualitative content analysis [49]. Second, the entire aggregated data set will be subjected to comprehensive multivariate analysis including treatment fidelity measured with the self-developed SEEGEN fidelity scale. The process evaluation will identify uptake of the intervention, and specity mediators and moderators of the effect.

### Participant timeline

Total trial duration is 24 months, consisting of an intervention phase (6 months) and a follow-up phase (3 months). In 2020, the workshops will be carried out in the intervention groups whereas the workshop for the CG will start after the follow-up phase. The CG will receive the same offer from the complex intervention. The different intervention modules vary in their duration: The first intervention module – *Top Management Training–* has a duration of 1 day (6 hours), the *Promoting Stress Preventive Relational Leadership Competence* has a total duration of 2 days (12 h) at intervals of 3 weeks. The third intervention module – *Dilemma Competency – Coping by Taking Responsibility –* has a duration of 2 days (12 h) at intervals of 4–8 weeks. The intervention module *Reconciling Work and Family Life* has a duration of one working day (6 hours) while *Stay Healthy at Work* is offered on 1.5 days over a period of 4 weeks (10 h). Booster sessions and the 1st-order round tables will take place within the first 6 months after T0, the 2nd-order round tables will be held after T1.

#### Preliminary timeline

**Table:** 

Total study duration	[24 months]
Process evaluation:	[10 months]
Duration of longest intervention:	[6 months]
Duration of whole intervention and observation phase:	[9 months]
Beginning of the preparation phase:	[11/2018]
Start of recruitment:	[10/2019]
End of recruitment:	[01/2020]
End of observation phase:	[12/2020]
Data base lock	[03/2021]
End of waitlist control intervention:	[04/2021]
Statistical analyses completed:	[08/2021]
Study report completed:	[09/2021]

#### Recruitment

Interested employees will be informed about the study and the different interventions by means of information events or by the operational health management of the respective location. Participation in the study is voluntary and consent may be withdrawn at any time without giving reasons or disadvantages. Verbal and written information will be given to all employees interested in the interventions.

### Methods: assignment of interventions

Allocation: For organisational reasons, cluster-randomisation will be carried out prior to recruitment of the first participants. The allocation will be done stratified by the three locations in a 1:1 fashion using a randomisation list which will be prepared by the Institute of Medical Biometry and Informatics (IMBI), University Hospital of Heidelberg, Germany. If appropriate, cluster pairs per site will be defined in order to prevent that matched pairs will be randomised into the same group.

Blinding: Due to the nature of the intervention blocks, blinding of participants and trainers is not possible.

### Methods: data collection, management, and analysis

#### Data collection methods

Individual participant data (sociodemographics and questionnaires on individual level) will be collected at T0, T1 and T2 (starting end of 2019) either paper-based (case report form, CRF) or web based electronically (electronical case report form, eCRF) in order to meet individual preferences.

Organisational indicators (on hospital/clinic level, assigned to clusters) will be collected either paper-based or electronically as well. Paper-based questionnaires will be sent to the IMBI by post. Data will be captured via double data entry near term to receipt and it will be transferred to a statistical analysis system running on a server located in the IMBI subsequently to data capture. Data captured in the eCRF will be transferred to the analysis system as well. The electronic data capture system (EDC-system) underlying the eCRF is also running on a server located in the IMBI. Data related to process evaluation will be collected and stored separately.

#### Data management

The system used for web based electronic data capture (EDC-system) is validated and compliant with FDA 21 CRF part 11. Data transmission is encrypted with secure socket layer (SSL) technology. The database server in the IMBI is located in a secure data centre and protected by a firewall. The system provides an infrastructure to support user roles and rights.

Only authorized users are able to enter or edit data. The access to centre specific organisational data is restricted to persons of the respective centre. Access to individual data is restricted to the participant only. All changes to data are logged with a computerized timestamp in an audit trail within the EDC-system.

Any modification of data captured from paper-based questionnaires via double data entry (e.g. as result of queries) will be logged within the analysis system as well. All individual data will be captured, transferred and stored in a pseudonymised manner. Backups of both, EDC-system and the statistical analysis system are conducted regularly.

High quality of data will be guaranteed by checking for completeness, consistency and plausibility on or near term to data entry. This will be realised by implementation of programmed validation rules, predefined in a data validation plan. The validation programs will generate queries/edit checks on data entry within the EDC-System and/or subsequent to data transfer to the analysis system by generating paper-based queries. The investigators or the designated representatives are obliged to clarify or explain the edit-checks and queries.

During study conduct, the database is accessible to the data manager and data entry staff only. After database closure, access rights will be granted to the responsible biometricians as well. Data will be managed and analysed according to the corresponding Standard Operating Procedures (SOPs) valid in the IMBI.

Organisational indicators (on hospital/clinic level) will be transferred to the Chair of Business Administration, in particular Work, Human Resource Management and Organisation Studies, of the Heinrich-Heine-University Düsseldorf for analyses. Study data and study documents stored in the IMBI will be transferred to the study coordinator subsequent to statistical report and/or publication for archiving.

#### Statistical methods

The primary analysis tests the null hypothesis that the absolute change in the total score of the IRR from T0 to T1 in the intervention group is equal to the absolute change in the total score in the control group. The analysis of the primary endpoint will be based on an ANCOVA model comparing the treatment groups including the baseline value of the total score of the IRR at T0 (baseline) with the total score of the IRR at T1 (follow-up) between intervention and control group, including hierarchy level (top management, middle management and employees without management responsibility) and gender (female, male, divers) as well as the site (planned are three) as covariates and the respective cluster as random effect. In case of significance, the WHO-5 will be tested in hierarchical manner in the same way. Again, in case of significance, the PSC-12 will be tested. Applying this hierarchical testing strategy, the overall type I error will be controlled. All other analyses will be of exploratory nature and interpreted only in descriptive manner. The primary analysis will be conducted based on the full analysis set reflecting the intention to treat principle including all participants in the group the respective cluster was randomised to. As sensitivity analyses, the per-protocol population (only participants which fulfil the inclusion criteria) will be analysed. There, only participants of the IG who participated in at least one session of a module are considered in the IG. Otherwise, these participants will be excluded from the analysis of the per-protocol population. The participation of individuals from the IG in further programs (roundtables and booster sessions) will be considered as additional covariate in further analyses of primary and secondary endpoints.

The analysis of the secondary endpoints within the population of cluster participants will be based on an ANCOVA model as well, comparing the cluster participants including the baseline value of the total score of the IRR, WHO-5, PSC-12 or single items at T0, respectively, with the total score of the IRR, WHO-5, PSC-12 or single items at T1, respectively, between cluster participants of a cluster-randomised into IG and cluster participants of a cluster-randomised into CG, including hierarchy level (top management, middle management, employees without management responsibility) and gender (female, male) as well as the site (planned are three) as covariates and the respective cluster as random effect. As additional secondary analyses, changes in the collected scores from T0 to T2 will be analysed.

Additionally, organisational indicators will be analysed using an ANOVA. The baseline value of scores of the organisational indicators, assigned to the intervention clusters, will be compared to the intervention clusters’ scores of the organisational indicators during later times of the intervention (with points in time preferably close to T0 (baseline), T1, and T2, but depending on the availability of the data in the accounting divisions), and examined for changes. Differences between the organisational indicators assigned to the intervention clusters and the organisational indicators assigned to the control group will be examined, respectively.

All data of clusters and participants will be described dependent on treatment group. Categorical data will be presented as frequencies and percentages. For continuous data, the number, mean, standard deviation, median, inter-quartile range, minimum and maximum will be calculated.

For further evaluation of potential factors influencing treatment effect, moderator and mediation analyses as well as subgroup analyses will be conducted. As example, the subgroup of a specific level of hierarchy will be evaluated. A detailed description of the planned statistical analyses will be provided in the Statistical Analysis Plan (SAP), which will be finalized prior to database closure and any analysis. All analyses will be conducted using SAS 9.4 or higher. Organisational indicators will be analysed using IBM SPSS Statistics 26 or higher.

### Handling of missing and spurious data and drop outs

If the IRR, WHO-5 or PSC-12 is missing at T0 or T1 for a participant due to loss to follow-up or other reasons, analyses will be done replacing the missing follow-up value on item level with the predictive mean matching method [50]. As sensitivity analysis, the baseline value will be carried forward resulting in no change for the primary endpoint. In addition, further sensitivity analysis will be conducted to evaluate the robustness of the results. If the complete IRR, WHO-5 or PSC-12 is missing at T0 and T1, the participants will be excluded for primary analyses according to a modified intention to treat principle.

### Method monitoring

#### Data monitoring

No data monitoring board will be established. The local management of each study site takes care of the security and quality of the data; as a confidence-building measure will serve 1) the clinic’s own data protection officers and the staff representatives 2) the independence of the university and its control by the ethics committees 3) medical confidentiality.

#### Harms

This trial has been assessed as low risk. No adverse events are being collected due to the type and content of intervention modules and the potential for this to create unnecessary burden on participants. In case of a specific psychological vulnerability of a participant, the trainer will offer advice at the end of the intervention module.

#### Auditing

Auditing is not intended. The local management of each study site takes care of the security and quality of the data. During study conduct, the database is accessible to the data manager and data entry staff only. After database closure, access rights will be granted to the responsible biometricians as well. Data will be managed and analysed according to the corresponding SOPs valid in the IMBI.

### Ethics and dissemination

#### Consent or assent

Verbal and written information will be given to all employees interested in the interventions. The study staff will contact potential participants and obtain written informed consent.

#### Confidentiality

To maintain confidentiality, a pseudonomised identification coded number and year of birth only will identify all evaluation forms, reports and other records. All study records will be kept in a locked file cabinet and code sheets linking a participant’s name to a participant’s identification number will be stored separately in another locked file cabinet.

The storage, evaluation and transfer of study-related data are carried out in accordance with statutory provisions and requires the participant’s voluntary written informed consent before participating in the study. The participants agree that data collected may be recorded on questionnaires and electronic data carriers and processed without naming. In addition, the participants agree that an authorised person who is bound to secrecy (e.g. persons conducting audits) may inspect the personal data collected insofar as this is necessary for the review of the project.

#### Access to data

During study conduct, the database is accessible to the data manager and data entry staff only. After database closure, access rights will be granted to the responsible biometricians as well.

#### Ancillary and post-trial care

Not applicable.

#### Dissemination policy

Study results will be disseminated widely through academic, policy and community networks and to trial participants. Beyond academic distribution, it will be especially important to share the data with health insurances and politics.

## Discussion

There seems to be growing psychological strain on people working in the health care sector worldwide. Impaired well-being of physicians burnout is nowadays generally regarded as a public health crisis in many high income countries, which also threatens patients´ care and safety [51]. For instance, surgeons’ burnout prevalence is clearly increased compared with the general population (53% vs. 28%) for multifactorial reasons [52]. Similarly, studies also consistently report a very high prevalence of hospital nurses in various disciplines that suffer from impaired mental well-being [53, 54].

Clinical, practical day-to-day experience, empirical evidence as well as written statements conclude compellingly that it will not be sufficient to address mental health phenomena on an individual level. It is concluded that tackling this issue needs – beyond individual and organisational interventions – the combined effort of administrative leadership of medical institutions, professional associations and politics [52]. In other words, a health system reform should be considered [51] addressing specific, often deteriorating working conditions for hospital staff. Therefore, the discussion of our major findings with key stakeholders of the German health system will be an important part of the project. Apart from discussing the necessary frameworks for an effective and systematic workplace health management in German hospitals, it will also be a matter of sensitising those stakeholders for the effects of public hospital financing on workload and thus on the mental health of health care employees.

Particularly, because of the increased financial pressure in the health care sector, it will be important for the sustainable implementation of the proposed complex intervention in German hospitals also to demonstrate its effects on economically relevant indicators, like absence rate, employer attractiveness, or intention to leave. In particular, staff that is urgently needed may leave a hospital and start to work elsewhere. In addition, it seems that working within the health care sector is perceived as less attractive. All these factors, and many more, may add to staff shortage and to the possible development of a somehow vicious cycle within the health care system.

Somehow surprisingly, it seems to be particularly difficult to implement structured and complex, i.e. behavioural as well as organisational health promotion programs especially for employees within the health care sector. This may be due to decreased awareness of psychosocial safety climate among leaders as well as among employees. Moreover, staff shortage and increasing cost cutting measures, problems in the interprofessional cooperation and time pressure might hinder the participation in interventions as well as the implementation of work design measures. In hospital settings, there is therefore often the paradox that participation in intervention measures is very difficult due to the staff shortage. This means that those who need the intervention most often have the least chance of participating in interventions. In order to implement an intervention like the one presented here, it is essential to convince all stakeholders in an organisation - in this case economical, medical (doctors and nurses) leaders as well as staff representatives in the hospital - and to provide the necessary resources for the organisation of the intervention.

In the planned study, a key success factor will be therefore to coordinate the intervention closely with the hospital work organisation (e.g. rostering) through close consultation with hospital managers and occupational health and safety professionals in order to achieve the desired participation rate. From an organisational prevention perspective, recent studies point out the importance of the active support of managers and executives to implement health related work design measures [55]. For example, managers provide the necessary personal, financial, and time-related resources, and have to agree to the implementation of work design measures. It is therefore essential to “prepare the field” and to sensitize managers for the importance of work design for the mental health of hospital employees. Thus, in preparing this cluster-randomised trial, a longstanding trustful relationship has been established with the higher management of the respective clinical centres.

In sum, supporting hospital staff in paying attention to their own health, in implementing constructive interpersonal and interprofessional working relationships, as well as using their practical knowledge and experience to improve organisational working conditions seems essential. And to support these professional groups as well as managers in charge of hospital administration to communicate their needs to health insurances and politics, who shape basic working conditions in the hospitals. Our complex intervention, evaluated by the planned trial, aims at providing qualitative as well as quantitative arguments to strengthen this approach.

## Data Availability

Data will be made available from the corresponding author on reasonable request.

## Abbreviations

BMBF: Bundesministerium für Bildung und Forschung [German Federal Ministry of Education and Research]
CG: Waitlist control group
CRF: Case Report Form
DRKS: Deutsches Register Klinikscher Studien [German Clinical Trial Register]
eCRF: Electronical Case Report Form
EDC: Electronic data capture
ERI: Effort-Reward Imbalance Scale
FIF: Fragebogen zur integrativen Führung [Questionnaire for integrative leadership]
GTL: Global Transformational Leadership Scale
HCS: Health and Social Services
ICC: Intraclass Correlation Coefficient
IG: Intervention group
IMBI: Institute of Medical Biometry and Informatics
IRR: Irritation Scale
LMX: Leader – Member-Exchange
PSC: Psychosocial Safety Climate
SAP: Statistical Analysis Plan
SEEGEN: SEElische GEsundheit am Arbeitsplatz KrankeNhaus [Mental health in the workplace hospital]
SOC: Selection, Optimization and Compensation
SOP: Standard Operating Procedure
SOSES: Short version of the Occupational Self-Efficacy Scale
SSL: Secure Socket Layer
TAA-KH-S: Tätigkeits- und Arbeitsanalyseverfahren für das Krankenhaus [Work Analysis Instrument for Hospitals]
WHO-5: World-Health Organization Well-Being Index
